# *Peste des petits ruminants* cases in Nigeria: A 10-year retrospective study from 2015 to 2024

**DOI:** 10.4102/ojvr.v92i1.2240

**Published:** 2025-11-21

**Authors:** Deborah A. Adetunji, Oluwaseun A. Ogundijo, Ahmad I. Al-Mustapha, Habiba Momoh, John O. Emethilue, Oluwakemi A. Osunderu, Veronica E. Adetunji, Emmanuel J. Awosanya

**Affiliations:** 1 Department of Veterinary Services, Ministry of Agriculture and Food Security, Ogun State, Nigeria; 2 Department of Veterinary Public Health and Preventive Medicine, Faculty of Veterinary Medicine, University of Ibadan, Ibadan, Nigeria; 3 College of Veterinary Surgeons of Nigeria, Abuja, Nigeria; 4 Department of Veterinary Services, Kwara State Ministry of Livestock Development, Ilorin, Nigeria; 5 Department of Food Hygiene and Environmental Health, Faculty of Veterinary Medicine, University of Helsinki, Helsinki, Finland; 6 Federal College of Animal Health and Production Technology, National Veterinary Research Institute, Vom, Nigeria; 7 Department of Veterinary Services, Ministry of Agriculture and Natural Resources, Asaba, Nigeria; 8 Research Coordinating Unit, Forestry Research Institute of Nigeria, Ibadan, Nigeria

**Keywords:** *Peste des petits ruminants*, spatio-temporal, trend analysis, prevalence, TADs, Nigeria

## Abstract

*Peste des petits ruminants* (PPR) is an endemic, transboundary animal disease (TAD) that affects small ruminants and threatens Nigeria’s food and economic security. This study evaluated the PPR cases in Nigeria using data from the World Animal Health Information System (WAHIS). Secondary data of PPR (2015-2024) were analysed as descriptive statistics, and we forecasted PPR cases from 2025 to 2030. Our findings showed temporal variations in PPR reports and case trends, with more cases (reports) between 2020 and 2024. There were significant variations in PPR cases across the six agro-ecological zones of Nigeria (*p* = 0.025) and across the states (*p* < 0.001). Bauchi (27.6%, *n* = 19 557/70 827) had over one-quarter of all national disease load, and Katsina had 9.6% (*n* = 6767/70 827) of the national PPR cases over the study period. Seven states had less than 50 cases, with Lagos and Ogun states having the lowest reports of PPR with 19 and 9 cases, respectively. Trend analysis forecasted an annual increase in the number of PPR cases, with at least 20 048 PPR cases in 2025, 22 838 cases in 2026, 25 737 cases in 2027, 28 745 cases in 2028, 31 860 cases in 2029, with the estimated number of cases rising to approximately 35 085 cases by 2030. The average case fatality rate for the 10 years was 14.1%. Hence, we estimated that at least 23 168 animals will be lost to PPR over the forecasted period.

**Contribution:**

The study emphasises the need for region-specific surveillance, targeted vaccination strategies, and predictive modelling tools for effective control and eradication efforts.

## Introduction

*Peste des petits ruminants* (PPR) is an endemic, viral disease of small ruminants that has significantly impacted the livestock sectors of many African and Asian nations. Its prevalence is impacted by things like wildlife interactions, trade activities and animal transportation (Mantip et al. 2022). The disease has a major effect on rural livelihoods, especially for smallholder farmers who depend on sheep and goats for both food security and revenue (Fayyad & Alzuheir 2024). Globally, the burden of PPR results in yearly economic losses that were estimated to be between $1.45 bn and $2.1 bn (Aboah et al. 2024; FAO & OIE 2016). In Africa, economic losses were estimated at $25 million annually (Meyer et al. 2025). In Nigeria, endemic PPR was estimated to cost livestock farmers some $10.4 m in annual losses (Esonu et al. 2022).

*Peste des petits ruminants* is a notifiable and transboundary animal disease (TAD) owing to its high rates of morbidity, mortality and the ability to cross international borders (Fathelrahman et al. 2021). The degree of PPR virus transmission in animal populations remains unknown, despite the existence of an effective vaccination that offers lifetime protection (Fournié et al. 2018). Aerosols between animals in close proximity are the main way that PPR infections are spread, and confinement can exacerbate outbreaks. Other sources of PPR introduction into a herd include: contaminated feed or water, and infection can originate from secretions and excretions of sick animals during incubation (Mantip, Shamaki & Farougou 2019).

*Peste des petits ruminants* is a vaccine-preventable disease (VPD). Despite the availability of a relatively cheap ($0.1 per dose), in-country produced effective vaccine, there are no robust (funded) national, regional or state-wide mass vaccination campaigns that cover a significant proportion of the small ruminant population in the country. Hence, the disease continues to cause significant losses of livelihoods, especially among rural women (owners of small ruminants), and threatens several sustainable development goals. To control endemic diseases like PPR, it is essential to regularly investigate spatio-temporal trends, risk factors and disease transmission dynamics to safeguard the livelihoods of livestock owners (Nkamwesiga et al. 2023). Hence, the objective of our study was to evaluate the spatio-temporal distribution and trends of PPR between 2015 and 2024 and forecast PPR cases between 2025 and 2030.

## Research methods and design

### Study design and data source

This study was conducted as an observational study of open access records to evaluate the number of PPR cases in Nigeria over the last 10 years (January 2015 – December 2024). The data were extracted from the World Animal Health Information System (WAHIS) https://wahis.woah.org, maintained by the World Organisation for Animal Health (WOAH). Our data focused on officially reported PPR outbreaks in Nigeria. The data are reported to the WAHIS by the Federal Government of Nigeria. At the national level, these PPR reports are curated from the monthly disease reports across all states in addition to laboratory confirmed cases (where feasible) by the National Veterinary Research Institute, Vom, Plateau State, Nigeria.

### Data analysis

The data were analysed using Statistical Package for Social Sciences (SPSS) version 29.0 and presented as descriptive statistics. The qualitative data were presented as proportions and figures, whereas the quantitative data were summarised as means and standard deviations. *Trend analysis was performed* using time series plots. The PPR heat map was generated using QGIS version 3.38. We used analyses of variance (ANOVA) to test for the statistical differences in the total number of PPR cases across the 36 states, including the Federal Capital Territory (FCT), and over the 10 years. Furthermore, we forecasted the number of PPR cases in Nigeria for the next 6 years (2025–2030) using the trend analysis tool (Holt’s model). This model (Holt’s Linear Trend Exponential Smoothing) was selected as it conducts exponential smoothing and explicitly captures disease trends based on the assumption that the trend is linear and persistent into the future (i.e. the rate of increase or decrease continues).

### Ethical considerations

The ethical clearance to conduct this study was obtained from the Ogun State Ministry of Agriculture and Food Security, Ijebu Ode, Ogun State, Nigeria with reference number: MOA/DVS/IJB/25/09/07/25. All retrieved data used were secondary and publicly available at the WAHIS database.

## Results

### Prevalence of *peste des petits ruminants*

**FIGURE 1:**
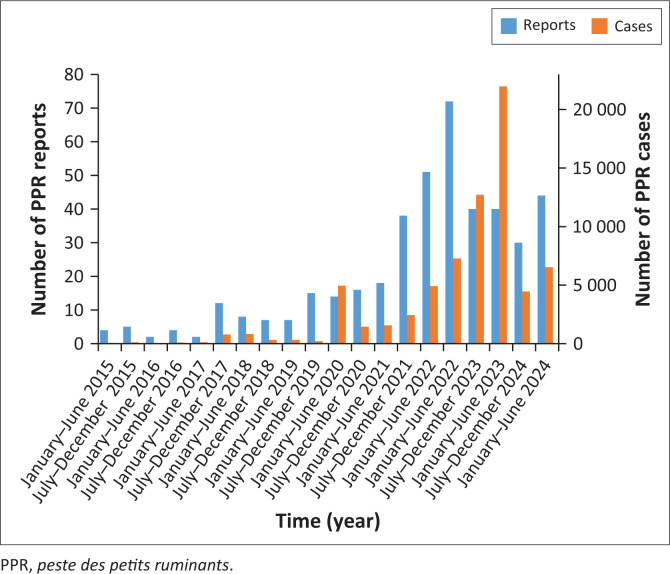
Temporal variations of *peste des petits ruminants* reports and cases in Nigeria (2015–2024).

There were a total of 422 reports of confirmed PPR outbreaks during the period under review. These reports represented 70 827 PPR cases, which were reported from the 36 states and the Federal Capital Territory between 2015 and 2024 (Figure 1). During the timeframe, the number of PPR in goats reported were 37 260, sheep 2758, sheep/goat (mixed herds) 30 801, and cattle 8. Our analysis also revealed significant temporal variations in both the number of PPR reports and the outbreaks across the country (*p* = 0.009) (Figure 1).

### Spatial variations in *peste des petits ruminants* cases

Approximately a quarter of all PPR reports were from the North Central region (23.7%, *n* = 100/422). There were a similar number of these reports among states in the South West (20.9%, *n* = 88/422), North West (20.6%, *n* = 87/422) and North East (18.5%, *n* = 78/422). States in the South South (8.7%, *n* = 37/422) and southeast (7.6%, *n* = 32/422) had the least number of PPR reports (Table 1). There were also significant variations in the number of confirmed PPR cases and case fatality rates (CFR) across the regions. Our findings revealed significant differences in the number of PPR reports across the six geopolitical zones in Nigeria (*p* = 0.0025).

**TABLE 1:** Distribution of *peste des petits ruminants* cases per region in Nigeria (2015–2024).

Geopolitical zones	Number of PPR reports	Number of PPR cases								
		Cases (*n*)					Case fatality rate (%)			
		< 50	51–500	501–999	1000–2000	> 2000	< 10	10–25	25–50	> 50
North Central	100	0	3	2	0	2	2	4	1	0
North East	78	1	1	1	1	2	3	3	0	0
North West	87	0	1	2	0	4	2	3	1	1
South East	32	2	2	0	1	0	0	1	3	1
South South	37	3	3	1	0	0	2	2	3	0
South West	88	1	1	0	2	1	2	3	0	0

PPR, *peste des petits ruminants*.

**FIGURE 2:**
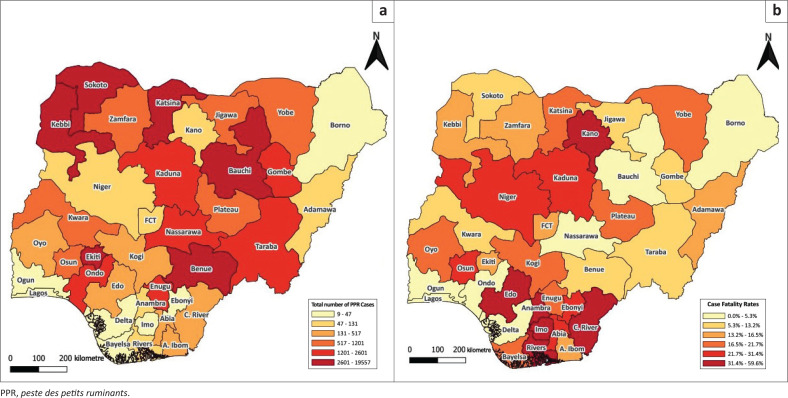
The heat maps showing (a) total cases, and (b) case fatality rates, of *peste des petits ruminants* in Nigeria (2015–2024).

There was marked regional variation in the spatial distribution of PPR cases in Nigeria from 2015 to 2024. When the regions were declustered into states, our findings revealed that there were significant differences in the reported cases of PPR across the 37 administrative divisions (states) of the country (*p* < 0.001). Two North Eastern states are emerging PPR hotspots as they had the highest number of PPR cases: Bauchi (27.6%, *n* = 19 557/70 827) had over one-quarter of all national PPR cases and Katsina had 9.6% (*n* = 6767/70 827) of the national PPR cases over the study period. Seven states had fewer than 50 cases over the 10-year period. Among the states with the lowest number of cases, Ebonyi (Southeast) and Ogun (Southwest) had the lowest report of PPR, with 18 and 9 cases, respectively (Appendix 1, Table 1-A1; Figure 2).

Figure 2 further showed that the case fatality rate was notably high in Imo (59.0%), Kano (58.3%), Anambra (50.0%), Edo (46.5%) and Cross River (45.6%), majority located in southern Nigeria. The least CFR of 0.0% was recorded in Ogun and Delta states. Some states, such as Bauchi, Benue and Sokoto, had high case burden, with very low CFR, while others like Imo, Kano and Anambra had higher CFRs, but lower burden of PPR (Appendix 1, Table 1-A1).

### Projected cases of *peste des petits ruminants* by 2030

**FIGURE 3:**
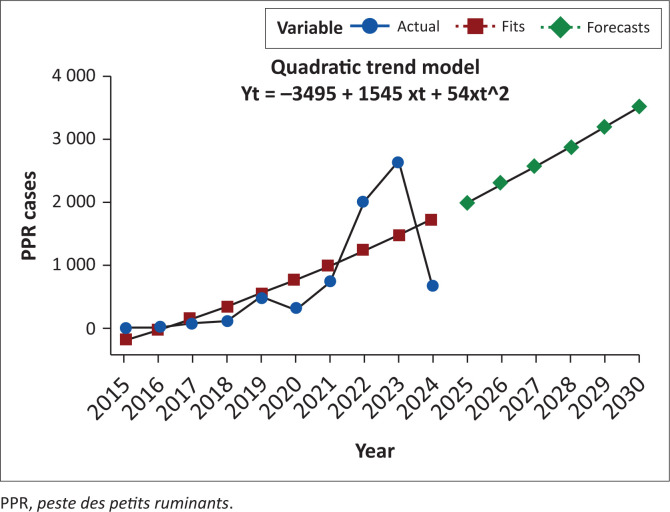
Forecast of *peste des petits ruminants* cases in Nigeria by 2030.

With the global target to significantly reduce the burden of PPR, we estimated the expected number of PPR cases in Nigeria between 2025 and 2030 (6 years) and estimated the annual mortality if no interventions were instituted. Our analysis revealed an increasing number of PPR cases unless a national comprehensive vaccination is implemented. Our model revealed that there would be at least 20 048 PPR cases in 2025, 22 838 cases in 2026, 25 737 cases in 2027, 28 745 cases in 2028 and 31 860 cases in 2029, with the estimated number of cases rising to approximately 35 085 by 2030 (Table 2; Figure 3). The average CFR for the 10-year period was 14.1%. Hence, we estimated that at least 23 168 animals will be lost to PPR over the forecasted period.

**TABLE 2:** *Peste des petits ruminants* cases in Nigeria, 2015–2024 and projections of *peste des petits ruminants* cases, 2025–2030.

Year	Number of susceptible animals	Number of PPR cases	Number of PPR deaths	Case positivity rate (%)	Case fatality rate (CFR)
2015	823	178	10	21.6	5.6
2016	868	134	26	15.4	19.4
2017	35 017	893	141	2.6	15.8
2018	19 734	1152	207	5.8	17.96
2019	31 668	5158	1174	16.3	22.8
2020	25 642	3006	374	11.7	12.4
2021	38 375	7353	1407	19.2	19.1
2022	68 334	19 994	2085	29.3	10.4
2023	92 978	26 432	2098	28.4	7.9
2024	17 228	6527	618	37.9	9.5
2025	-	20 048†	2827†	-	-
2026	-	22 838†	3220†	-	-
2027	-	25 737†	3629†	-	-
2028	-	28 745†	4053†	-	-
2029	-	31 860†	4492†	-	-
2030	-	35 085†	4947†	-	-

PPR, *peste des petits ruminants*.

† , Forecasted PPR cases and associated mortality.

## Discussion

The high prevalence of PPR in Nigeria causes productivity losses in sheep and goats, which are crucial assets for rural households, especially women and smallholder farmers and poses significant barrier to the attainment of the Sustainable Development Goals (SDGs), particularly those related to poverty reduction, zero hunger and sustainable livelihoods (OIE 2022). This persistent burden undermines income generation, nutrition and resilience in vulnerable communities, slowing progress towards SDG 1 (No Poverty), SDG 2 (Zero Hunger) and SDG 8 (Decent Work and Economic Growth). Moreover, Nigeria’s struggle to effectively control PPR represents a missed opportunity for the Global PPR Control and Eradication Strategy, which aimed for the eradication of the disease by 2030.

This study revealed that there were significant temporal variations in PPR reports and outbreaks observed in Nigeria. Our findings revealed low, but stable, PPR reports *from 2015 to mid-2019, averaging* fewer than 10 reports per half-year. This agrees with a study that reported that PPR reporting was comparatively low in various regions in Africa (Banyard et al. 2010). From *late 2019 to 2021, the* reports began to rise gradually, which indicates increased surveillance or the early onset of outbreaks. In *2022, the reports peaked*, showing that a significant surge occurred, with the highest number of reports (approximately 72) recorded in *January 2022 – June 2022*. This may reflect intensified surveillance or widespread outbreaks. *However, from 2023 to mid-2024, there is* a slight decline that followed the 2022 peak, although the number of reports remained substantially higher than pre-2020 levels. This suggests a new baseline or ongoing monitoring in response to elevated risk. This aligned with the report of Chabiri et al. (2023). These slight differences shown could be because of their study design, in which they focused only on reports of suspected cases sent to them, and WAHIS data included epidemiologically linked cases. In addition, climate change, disease surveillance initiatives, and animal movement and commerce were probably some of the variables influencing these temporal fluctuations (Balamurugan et al. 2021).

*The number of confirmed PPR cases* followed the same pattern as the reports, with low, stable reports between 2015 and 2019, peaking in 2022, and a slight drop in 2024. This could suggest the onset of larger or renewed outbreaks and expanded surveillance efforts (Imanbayeva et al. 2025). The introduction of intensified control programmes and improved diagnostic capabilities could contribute to a better understanding of disease events in endemic regions (OIE & FAO 2015). A steady rise was seen through 2021 and 2022. In *2023, which was the peak period*, the number of cases surged dramatically, peaking at over 21 000 in *January 2023 – June 2023* – the highest recorded in the timeline. This points to either a massive outbreak or major improvements in detection or reporting, and surveillance. In *2024*, PPR cases declined but remained high compared to earlier years, indicating a sustained endemic presence. The decline in 2024, despite still reflecting a high case load, matches common epidemiological patterns where initial control measures begin to impact the disease cases (burden). These findings align with common epidemiological patterns observed during outbreak interventions (Lawrence 2024). After such peaks, especially after major interventions, a decline is often observed, although disease persistence indicates an ongoing endemic presence. This correlates with a similar study (Adombi et al. 2017) in the Republic of Benin, which proved that despite control efforts, *peste des petits ruminants* virus has persisted in Benin since the 1960s. These changes likely explain the significant rise in case numbers rather than a sudden expansion of the disease (Parida et al. 2016).

There were significant differences in the number of PPR cases among the 37 states, with states like Bauchi, Kebbi and Katsina having the highest cases and states like Ebonyi and Ogun having the lowest number of cases. These differences in the number of PPR cases could be because of the substantially different prevalence of PPR across the different agroecological zones of the country. In addition, Bello et al. (2016) reported that PPR seroprevalence among sheep and goats in Bauchi was 60.4%. Also, Nkamwesiga et al. (2023) highlighted that communal grazing, transhumance practices and restricted access to veterinary care are some of the causes of the high prevalence of PPR in Bauchi State. These actions make it easier for PPR to spread among vulnerable animal populations (Walle et al. 2024). In Dutsin-Ma Local Government Area of Katsina State, the antibodies against PPR were detected in 40% of small ruminants (Tarnongo, Egbo & Kalla 2022). The report is in consonance with our findings and emphasised the necessity of more aggressive control measures and vaccination efforts because of the endemic nature of PPR in the region. In contrast to states with a high number of cases, Ebonyi State has a lower number of PPR cases. This result is in consonance with the report of Chukwudi et al. (2020), which found a seroprevalence rate of 20.3% in Ebonyi, exhibiting a low PPR burden, which was lower than neighbouring states like Enugu and Anambra. The nine PPR cases from Ogun State are inconsistent with the findings of the study of Okwelum et al. (2021) in Abeokuta, which reported PPR outbreaks with morbidity rates of 80% – 100% and mortality rates ranging from 10% to 70%. Therefore, the Ogun State veterinary services must be supported to improve the sensitivity of its PPR surveillance system, the timeliness of reports, and implement massive vaccination campaign strategies.

The spatial analysis of the case distribution from 2015 to 2024 revealed the concentration of PPR in emerging hotspot states such as Bauchi and Kebbi. The regional locations of these areas are characterised by dense small ruminant populations and transhumance pastoral systems, which may have encouraged transmission of the disease (Maikano et al. 2020). However, some of the high burden states such as Bauchi, Benue, Nassarawa and Sokoto, had low CFRs, while some other low-incidence states, such as Imo, Anambra and Kano, had disproportionately high CFRs. These may have resulted from potential gaps in early detection, clinical management or under-reporting of mild cases (FAO & WOAH 2022). These findings reinforced the need for region-specific control strategies, combining intensified surveillance with targeted veterinary response and aligned with recent calls for integrated, spatially informed approaches to achieve the global PPR eradication goal by 2030. Hence, more vaccinations are needed in emerging hotspots such as Bauchi, Kebbi, Sokoto, Benue and Ekiti States.

The significant variation in PPR cases between states could be attributed to local context-specific factors, such as performance of veterinary services, livestock movement, vaccination coverage, environmental factors and socio-economic conditions, which are likely to influence the rate of PPR disease events (Chukwudi et al. 2020; Esonu et al. 2022). Higher PPR frequency was discovered in Pakistan’s rural areas, which may be comparable to state-level variations seen in Nigeria, where places with larger rural populations typically had higher exposure and less stringent control methods (Liu et al. 2018; Zafar et al. 2024). In areas where livestock are kept in large numbers and in close proximity, such as in the North Eastern states, outbreaks tend to spread more quickly, which could explain the regional differences (Bello 2013). These findings are also consistent with other scholarly articles on PPR in Nigeria and globally, where more pastoralist areas exhibit comparable trends, which highlight the role of local contexts in determining the spread and control of this disease. For instance, a study by Esonu et al. (2022) highlighted how differences in socio-economic status and farming practices across states in Nigeria contributed to varying PPR prevalence. Studies conducted in East Africa have also revealed notable regional variations in PPR prevalence, with nations such as Kenya (Kihu et al. 2015; Lugonzo et al. 2023; Omani et al. 2019) and Ethiopia (Agga et al. 2019; Kumbe et al. 2024; Reta 2024) citing high PPR incidence in pastoralist areas where livestock are regularly moved over long distances, making vaccination and control more difficult.

*Peste des petits ruminants* is a highly contagious viral disease that affects small ruminants, with mortality rates reaching up to 90% in infected populations (Kumar et al. 2014). Although there were more PPR cases in the northern states, the CFR was higher among states in southern Nigeria (Figure 2). This could be because of better case management associated with higher burden in the northern states or the lower exposure risk among small ruminant breeds (especially West African Dwarf) in southern Nigeria. With the global target to significantly reduce the burden of PPR, we estimated the expected number of PPR cases in Nigeria between 2025 and 2030 (6 years). Our analysis forecasted an increasing number of PPR outbreaks (cases) unless mass national vaccination campaigns are implemented. This finding was consistent with current trends and emphasised the need to implement a comprehensive intervention for the control of PPR (Imanbayeva et al. 2025). The primary PPR control strategy involves mass vaccination of significant small ruminant populations in endemic countries or regions (Fayyad & Alzuheir 2024; OIE & FAO 2015). Hence, the importance of effective vaccination programmes and strategic surveillance to achieve the global goal of eradicating PPR by 2030 cannot be over-emphasised. The projected increase in PPR cases is likely to occur without a comprehensive and sustained national vaccination strategy. The PPR estimates of the Holt’s model were similar to those of the quadratic trend model despite the latter being deterministic, sensitive to outliers, and can project sharp changes in disease incidence. Based on both models, significant economic losses await livestock owners as we expect more than 23 168 small ruminants will be lost (14.1% CFR) if interventions are not instituted.

In addition, Chabiri et al. (2023) provided data that indicated a persistent endemic situation, with projections of a linear increase in PPR cases if control measures are not intensified. International organisations have prioritised the progressive control and global eradication of PPR by 2030, focusing on mass vaccination of small ruminant populations in endemic regions or countries (Hammami et al. 2018). The improved prospects for PPR control can be achieved through coordinated interstate vaccination efforts (Gurrappanaidu et al. 2025). *Peste des petits ruminants* eradication would be more likely if vaccination plans were tailored to the unique features of the local epidemiological setting and small ruminant population dynamics. This would also optimise the use of scarce resources (FAO 2017; Fournié et al. 2018). Without strengthened surveillance, vaccination and regional coordination, Nigeria’s high PPR prevalence threatens not only its own development targets but also global efforts, as uncontrolled transmission within such a key livestock-producing country jeopardises the feasibility of achieving global eradication within the set timeline.

Despite the new information generated by our study, the findings of this study have the following limitations. Firstly, there was gross under-reporting, delays in outbreak notification and data gaps in certain states and years which may affect accuracy, precision of trend and spatial analysis, and the assumptions in the projection models, which could have underestimated the true number of cases of PPR in Nigeria. Secondly, our forecasts did not account for future interventions (such as mass vaccination campaigns), and the model did not include factors related to deaths or offtake. In addition, data from WAHIS rely on national veterinary services, which may have varying capacities across the nation. Despite these limitations, the findings provided important insights for shaping data-driven, location-specific PPR control strategies in Nigeria.

## Conclusion and recommendations

This study has provided critical insights into the dynamics of PPR outbreaks across Nigeria, revealing significant *temporal and spatial variations* in the number of PPR cases. The analysis showed distinct *trends in both reported cases and the number of cases* over the years, with evidence of *yearly and regional differences in PPR cases*. The significantly increasing number *of forecasted PPR cases points to* the urgent need for sustainable and data-driven disease control strategies. It is also important to emphasise the need for region-specific interventions to achieve effective disease control and eventual eradication. We therefore recommend targeted interventions focused on highly affected states and/or regions identified through spatial analysis, strengthening of active surveillance systems and the strategic vaccination campaigns based on seasonal and regional patterns, and investment in predictive modelling and Geographic Information System (GIS) tools for early warning and resource optimisation.

## Appendix 1

**TABLE 1-A1:** Distribution of *peste des petits ruminants* cases per state in Nigeria (2015–2024).

State	Number of cases	Number of PPR deaths	CFR (%)	CFR categorisation	Case-categorisation
Abia	186	52	28.0	25% – 50%	51–500
Adamawa	108	15	13.9	10% – 25%	51–500
Akwa ibom	517	72	13.9	10% – 25%	501–999
Anambra	60	30	50.0	25% – 50%	51–500
Bauchi	19557	702	3.6	< 10%	> 2000
Bayelsa	39	7	17.9	10% – 25%	< 50
Benue	5910	407	6.9	< 10%	> 2000
Borno	23	1	4.3	< 10%	< 50
Cross River	320	146	45.6	25% – 50%	51–500
Delta	43	0	0.0	< 10%	< 50
Ebonyi	18	5	27.8	25% – 50%	< 50
Edo	155	72	46.5	25% – 50%	51–500
Ekiti	4840	723	14.9	10% – 25%	> 2000
Enugu	1543	270	17.5	10% – 25%	1000–2000
FCT	121	20	16.5	10% – 25%	51–500
Gombe	1818	156	8.6	< 10%	1000–2000
Imo	47	28	59.6	> 50%	< 50
Jigawa	573	44	7.7	< 10%	501–999
Kaduna	2226	700	31.4	25% – 50%	> 2000
Kano	72	42	58.3	> 50%	51–500
Katsina	6767	1339	19.8	10% – 25%	>2000
Kebbi	11635	1769	15.2	10% – 25%	>2000
Kogi	369	80	21.7	10% – 25%	51–500
Kwara	751	99	13.2	10% – 25%	501–999
Lagos	19	1	5.3	< 10%	< 50
Nasarawa	2601	53	2.0	< 10%	> 2000
Niger	131	34	26.0	25% – 50%	51–500
Ogun	9	0	0.0	< 10%	< 50
Ondo	1594	62	3.9	< 10%	1000–2000
Osun	1201	278	23.1	10% – 25%	1000–2000
Oyo	170	33	19.4	10% – 25%	51–500
Plateau	598	121	20.2	10% – 25%	501–999
Rivers	69	23	33.3	25% – 50%	51–500
Sokoto	3412	295	8.6	< 10%	> 2000
Taraba	2137	252	11.8	10% – 25%	> 2000
Yobe	595	129	21.7	10% – 25%	501–999
Zamfara	593	80	13.5	10% – 25%	501–999

Note: Data were obtained from the WAHIS website.

WAHIS, World Animal Health Information System.
